# PDK2-enhanced glycolysis aggravates fibrosis via IL11 signaling pathway in Graves’ orbitopathy

**DOI:** 10.3389/fimmu.2025.1537365

**Published:** 2025-02-13

**Authors:** Zhiyu Peng, Rui Huang, Lu Gan, Jinghan Wang, Xiaofeng Li, Jie Ding, Yinan Han, Jihong Wu, Kang Xue, Jie Guo, Rui Zhang, Jiang Qian, Ruiqi Ma

**Affiliations:** ^1^ Department of Ophthalmology, Fudan Eye & ENT Hospital, Shanghai, China; ^2^ Laboratory of Myopia, Chinese Academy of Medical Sciences, Shanghai, China; ^3^ NHC Key Laboratory of Myopia, Fudan University, Shanghai, China; ^4^ Department of Ophthalmology, The First Affiliated Hospital, Zhejiang University School of Medicine, Hangzhou, Zhejiang, China

**Keywords:** glycolysis, interleukin-11, transforming growth factor β1, orbital fibroblast, Graves’ orbitopathy

## Abstract

**Objectives:**

Transforming growth factor β1 (TGFβ1)-interleukin 11 (IL11) is a newly found critical signaling pathway in fibrotic diseases such as Graves’ orbitopathy (GO). It has now been confirmed that enhanced glycolysis plays a key role in the pathogenesis of GO. However, little is known about the relationship between glycolysis and IL11-mediated fibrosis in GO. This study aimed to identify the relationship between glycolysis and TGFβ1-IL11 signaling pathway and investigate the role of IL11 in glycolysis-facilitated fibrosis in GO.

**Methods:**

Orbital connective tissues were collected from GO and control patients. Primary orbital fibroblasts (OFs) were cultured from clinical tissues. Patient-derived xenografts were established via intraorbital transplantation of GO orbital tissue in humanized NCG mice. Protein levels were measured using Capillary Western Immunoassay (WES). Small interfering RNA (siRNA) was used to construct transfected OF strains. Lactate production was measured to assess glycolysis status. Animal models were assessed by T2-weighted magnetic resonance (MR) scan. Immunohistochemistry staining was applied to patients’ orbital connective tissues.

**Results:**

Orbital connective tissues were collected from GO patients. Immunohistochemical (IHC) staining of GO tissues revealed the phenomenon of pyruvate dehydrogenase kinase 2 (PDK2)-enhanced glycolysis and upregulated IL11-IL11Rα pathway. *In vitro* experiments showed successful induction of fibrosis of patient-derived orbital fat/connective tissues, which could be alleviated by dichloroacetic acid (DCA). MRI images and analysis of hematoxylin and eosin (HE) and Masson-stained section demonstrated enhanced glycolysis in GO, facilitating fibrosis of the orbital tissue. Targeting PDK2 decreased IL11 expression to suppress fibrosis. *In vivo* experiment confirmed anti-fibrotic effect of inhibition of glycolysis.

**Conclusions:**

PDK2-enhanced glycolysis exacerbates fibrosis via IL11-IL11Rα signaling pathway, shedding light on a potential therapeutic role of metabolic modulators such as DCA in GO treatment.

## 1 Introduction

Graves’ orbitopathy (GO) is an autoimmune disorder of orbital tissues including extraocular muscles and is meanwhile the major extrathyroidal manifestation of Graves’ disease (GD) (1, 2). The orbital connective tissues undergo inflammatory infiltration, edema, enlargement, tissue remodeling, and finally fibrosis (3), which leads to exophthalmos, ocular bias, eye movement disorders, restrictive strabismus, and other complications (1, 3) and seriously impairs patients’ quality of life.

The orbital fibroblast (OF) is considered the key cell responsible for pathogenesis of GO (4). The orbital fibroblasts (OFs) differentiate into myofibroblasts under the stimulation of a variety of cytokines and express α smooth muscle actin (αSMA), which is used as a primary marker to detect this biological response (5). Many studies have identified differences between GO and control OFs in their ability to cope with oxidative stress and antioxidant (6–8). In our previous studies, we discovered enhanced glycolytic pathway in GO OFs via pyruvate dehydrogenase kinase 2 (PDK2) overexpression, which could be inhibited by the PDK inhibitor dichloroacetic acid (DCA) (9). Our further research demonstrated that glycolysis facilitated ferroptosis resistance in the GO OFs (10). Therefore, glycolysis may be one of the important pathogenic mechanisms of GO and has the potential to serve as a target for anti-fibrosis therapy.

Elevated transforming growth factor β1 (TGFβ1) signaling cascade has been confirmed a central driver during fibrosis in an array of fibrotic disease (11–13). Emerging evidence suggests that glycolysis affect TGFβ1 signaling, promoting the differentiation of fibroblasts into myofibroblasts and regulating fibrotic procedure in lung fibrosis (14, 15), obesity (16), and systemic sclerosis (17). However, due to this pathway’s extensive distribution and pleiotropic roles in normal life activities of cells, past attempts targeting TGFβ1 failed (18–20). Research therefore shifted focus to downstream. Interleukin 11 (IL11), a member of interleukin 6 (IL6) family, is an important downstream regulator of TGFβ1 pathway (21). IL11 can be secreted by fibroblasts, osteoblasts, endothelial cells, lung smooth muscle cells, and so forth (22). IL11 specifically binds to IL11 receptor alpha subunit (IL11Rα) and glycoprotein (gp)130 receptor (23). IL11Rα is highly expressed on fibroblasts (24). Congenital deletion of IL11 signaling does not cause fatal outcomes, making it a potential therapeutic target worth investigating (24, 25). *In vivo* and *in vitro* experiments have demonstrated that blocking IL11 signaling pathway can effectively play an antifibrotic role in many diseases (26–28). It has also been confirmed in GO that IL11 signaling plays a critical role in the phenotype switching of orbital fibroblasts (29). Based on these observations, we hypothesized that DCA may inhibit fibrosis in the GO OFs through suppress IL11 signaling.

In this study, we investigated PDK2, IL11, and IL11Rα levels in the orbital connective tissues of patients with GO compared to healthy controls. We further elucidated the effects on fibrotic features of glycolysis and TGFβ1-IL11 signaling pathways and investigated the role of IL11 in glycolysis-facilitated fibrosis.

## 2 Materials and methods

### 2.1 Materials

Reagents included Recombinant Human TGFβ1 (PeproTech, Cat# 100-21, Rocky Hill, NJ, USA), Human IL11 Recombinant Protein (Gibco, Cat# PHC0115, Grand Island, NY, USA), and PDK inhibitor dichloroacetic acid (DCA, Sigma-Aldrich, Cat# D54702, St. Louis, MO, USA). Commercial kits included Human IL11 DuoSet ELISA (R&D System, Cat# DY218, Minneapolis, MN, USA), Lactate ELISA kit (Abcam, Cat# ab65331, Cambridge, MA, USA), rtPCR kit (TAKARA, Cat# RR047, Tokyo, Japan), SYBR qPCR kit (TAKARA, Cat# RR820, Tokyo, Japan), RNAsimple Total RNA kit (TIANGEN, Cat# DP419, Beijing, China), and Capillary Western Immunoassay kit (ProteinSimple, 12-230 kDa, San Jose, CA, USA). Primary antibodies targeted αSMA (monoclonal, Abcam, Cat# ab7817, Cambridge, MA, USA), IL11Rα (monoclonal, Abcam, Cat# ab125015, Cambridge, MA, USA), IL11 (Abcam, Cat# ab187178, Cambridge, MA, USA), PDK2 (Abcam, Cat# ab68164, Cambridge, MA, USA), and GAPDH (CST, Cat# 2118, Danvers, MA, USA). Validations of the primary antibodies are provided on the manufacturer’s website.

### 2.2 Subject recruitment

Orbital fat/connective tissues and peripheral blood mononuclear cell (PBMC) were collected from 20 GO patients during orbital decompression surgery and from 10 control subjects during strabismus or orbital surgery. For GO subjects, the inclusion criteria were patients with extraocular muscle enlargement on CT/MRI scan and limited globe motility; the exclusion criteria were patients who received orbital radiotherapy or received effective systemic steroid therapy within 3 months. For control subjects, the inclusion criteria were patients with concomitant strabismus, cosmetic orbital decompression, enucleation due to trauma, or blepharoplasty; the exclusion criteria were patients with orbital inflammatory disease, thyroid dysfunction, orbital infection, or previous intraorbital malignant tumors. The baseline characteristics of the enrolled subjects are summarized in **Table 1**. Written informed consent was obtained from each subject, and the study protocol was approved by the Institutional Review Board of Fudan Eye and ENT Hospital.

**Table 1:** Clinical information of recruited subjects.

	GO (n=20)	Control (n=10)
Gender (male/female)	8/12	6/4
Age (years)	47.6 ± 13.4	41.0 ± 15.1
Clinical activity score^a^	1.3 ± 0.9	\
Duration of GO (months)	12.8 ± 10.6	\
Duration of GD (months)	42.4 ± 69.0	\
Thyroid function upon recruitment		
Euthyroid	19	10
Hyperthyroid	1	0
Hypothyroid	0	0
Therapy history		
Systemic steroid (within six months)	7	0
Radioactive iodine therapy	1	0
Thyroidectomy	0	0
Antithyroid treatment	16	0
Smoking status		
Current smoker	4	2
Previous smoker	0	1
Nonsmoker	16	7

a The clinical activity score was rated on a scale of 0-7 with the following seven items according to the 2021 EUGOGO Clinical Practice Guidelines: eyelid swelling, eyelid erythema, conjunctival redness, chemosis, caruncle or plical inflammation, spontaneous orbital pain, and gaze evoked orbital pain.

GO, Graves’ orbitopathy; GD, Graves’ disease.

### 2.3 Histological staining

The connective tissue was fixed with neutral formalin, embedded in paraffin, and sectioned in the sagittal or horizontal plane. The slides were processed with hematoxylin and eosin (HE) staining, Masson trichrome staining, PDK2, IL11, and IL11Rα immunohistochemical staining. Brightfield images were taken with a microscope (Leica Microsystems). ImageJ (version 1.54k) was used for calculating the region of interest (ROI). Threshold was adjusted manually to accord with connective tissue region and staining positive region, and the integrated option density (IOD) and the area of target protein distribution (area) could be obtained. Average optical density (AOD)=IOD/area.

### 2.4 Cell culture

Orbital connective tissue was minced into 5-mm^3^ pieces and placed in six-well format containing high glucose Dulbecco’s modified Eagle’s medium (DMEM) (Gibco, Cat# 11965, Grand Island, NY, USA) supplemented with 20% fetal bovine serum and 1% penicillin/streptomycin (Cytiva, Cat# SV30010, Wilmington, DE, USA). After reaching 90% confluence, the primary OFs were passaged with 0.25% trypsin (Gibco, Cat# 25200, Grand Island, NY, USA) and maintained in DMEM supplemented with 10% FBS and 1% penicillin/streptomycin. The cells were grown and maintained at 37°C with 5% CO_2_. The culture fluid was renewed every 2–3 days. All the experiments were carried out at low passage (P3–P8). Cells were serum starved for 16 h before stimulations.

### 2.5 Immunofluorescence staining

Primary OFs were seeded on glass coverslips at 8×10^3^ per well. Cells were fixed with 4% paraformaldehyde for 20 min, permeated with 0.5%Triton for 30 min, and blocked with 3% BSA for 1 h at room temperature. Cells were incubated with primary antibodies (1:200 anti-αSMA) at 4°C for 12–16 h. After extensive rinsing, coverslips were incubated with the Alexa Fluor 555-labeled secondary antibodies (Invitrogen, Cat. No. A-21422) in the dark at room temperature for 1 h and stained with DAPI (1:1,000) for 5 min. A confocal microscope (Leica TCS SP2; Leica Microsystems) was used for imaging. Cell morphological changes were quantitatively described by cell length–width ratio.

### 2.6 Enzyme-linked immunosorbent assays

Culture supernatant was collected and assayed according to the manufacturer’s protocol. The R&D IL11 ELISA kit was adapted to measure IL11 concentrations.

### 2.7 Lactate production assay

Primary OFs were seeded on a 15-cm dish. OFs were deprived of serum for 12 h after reaching 95% confluence and incubated with siRNA for 12 h. TGFβ1 (10ng/ml) ± DCA (5mM) were given in 1% FBS and harvested for intracellular lactate measurement after 12 h according to the instruction of Lactate ELISA kit.

### 2.8 Capillary Western Immunoassay

Protein samples were extracted from unfrozen cells with RIPA reagent (Beyotime, Cat. No. P00138), quantified using BCA protein assay kit (Beyotime, Cat. No. P0012), and immunodetected with a Western blot system (WES; ProteinSimple) according to the manufacturer’s protocol. The relative amount of each immunoreactive band was quantified by signal intensity and normalized to GAPDH in the same sample. Different loading concentration of protein samples was tested by a titration experiment. The suitable concentration was determined as 0.5 μg/μl for αSMA (1:10), IL11Rα (1:10), PDK2 (1:10), and GAPDH (1:50).

### 2.9 Reverse-transcription PCR

Total RNA was extracted using an RNAsimple Total RNA kit (Tiangen, Cat. No. DP419) and reverse transcribed to cDNA with a rtPCR kit (TAKARA, Cat. No. RR047).

### 2.10 Quantitative real-time PCR

Real-time quantitative PCR was performed on a CFX96 Real-Time System (C1000 Touch, Thermal Cycler) with the SYBR qPCR kit. Primer sequences used are shown in **Table 2**. The amplification efficiency was evaluated by the standard curve method. The mRNA level was normalized to *GAPDH* by the −ΔΔCT method.

**Table 2:** Primers for quantitative real-time PCR.

Gene	Primer (Forward 5’→3’)	Reverse (Forward 3’→5’)
*ACTA2*	CAGGGCTGTTTTCCCATCCAT	GCCATGTTCTATCGGGTACTT
*IL11*	ACAGCTGAGGGACAAATTCC	CCGCAGGTAGGACAGTAGGT
*IL11Ra*	GCCGACTATGAGAACTTC	ACTCCTCCTCTGGCTATC
*PDK2*	AACCTGCTTCCCGACCGAGT	TCCTCGGGATCCTTGTCCA
*GAPDH*	TGTTGCCATCAATGACCCCTT	CTCAGCCTTGACGGTGCCAT

### 2.11 siRNA transfection

Primary OFs were seeded in six-well plates to reach 80% confluence and transfected with Lipofectamine 3000 according to the manufacturer’s instruction. Briefly, after 6-h starvation, the OFs were treated with Opti-MEM (Gibco, Cat. No. 3198508) containing PBS (blank group), Lipofectamin 3000 + scramble siRNA (RIBOBIO, siN0000001; negative control group), Lipofectamin 3000 + *PDK2* siRNA (sense 5′→3′: GACCGAUGCUGUACUCUAUTT; antisense 5′→3′: AUAGAUGACAGCAUCGGUCTT), Lipofectamin 3000 + *IL11* siRNA (RIBOBIO, stB0006813B), and Lipofectamin 3000 + *GAPDH* siRNA (RIBOBIO, siP0000001; positive control group) for 24 h. The transfected cells were treated with other reagents for further experiments.

### 2.12 Generation of humanized NCG mice

Four- to six-week-old female NOD/ShiLtJGpt-Prkdcem26Cd52Il2rgem26Cd22/Gpt (NCG) mice purchased from GemPharmatech Laboratories were used in the xenograft experiments. NCG mice were preconditioned with irradiation (1.2 Gy). A total of 1×10^7^ human PBMC were transplanted intravenously into each mouse within 24 h after irradiation (30). Flow cytometry was performed to detect the proportion of human CD45^+^ cells in the peripheral blood after 3 weeks. Mice with over 25% hCD45^+^ cells were considered humanized NCG mice.

### 2.13 Orthotopic engraftment of GO orbital tissue

NCG mice were kept on a standard 12-h light–dark cycle. The human orbital tissue (fat and connective tissues, no eye muscle tissues) was obtained at orbital decompression surgery. These tissues were cut into small pieces of 1 × 1 × 1 mm^3^ and xenografted into orbit of mice at the same time with intravenous injection of PBMC. Mice were observed once a week and were sacrificed after 3 weeks. HE and Masson staining were performed on collected orbital connective tissues. Every 3 days, 500 mg/kg of DCA or PBS was administered by gavage.

### 2.14 Magnetic resonance imaging

A 7-T Bruker Clinscan animal MRI scanner (Bruker BioSpin MRI GmbG, Germany) equipped with a four-channel phase-array surface coil was used to examine orbital connective tissues of mice. Isoflurane were used to anesthetize the mice. The mice were placed on a dedicated mice scan bed (Bruker, Ettlingen, Germany) and T2-weighted TurboRARE sequence scanning (TR/TEeff = 2,700/30 ms, RARE factor = 8, NA = 12, spatial resolution = 0.62 mm × 0.62 mm × 0.3 mm, slices = 7, no gaps, scan time 16 min) was applied to them. Two independent observers (observer 1 had 5 years of experience in orbital radiology; observer 2 had 3 years of experience in orbital radiology) manually delineate ROI. Two small circular ROI (0.09–0.10 mm^2^) were placed in extraocular muscle region or on the ipsilateral white matter of brain as the calibration function. Signal intensity of orbital fat and brain was measured, and the ratio was obtained as “OD_Orbital Fat/Brain_/OS_Orbital Fat/Brain_.” Take the ratio between the right eye and left eye and each mouse’s tissue-specific signal intensity ratio (Signal Intensity_Orbital Fat/Brain_) was acquired.

### 2.15 Statistics

Statistical software was GraphPad Prism 8.0.2 (version 26.0). All continuous variables with normal distribution were shown as mean ± standard error of mean (SEM). Shapiro–Wilk test was applied to assess normality of data. Student’s t-test or Welch’s t-test was applied to compare two independent experimental groups. Paired t-test was applied to compare data from GO or control OFs. Pearson correlation and linear regression were applied to analyze correlation between two continuous variables. Statistical significance was defined as *p*-value < 0.05.

## 3 Results

### 3.1 Glycolysis exhibits positive correlation with IL11 pathway in GO patients

**Figure 1:**
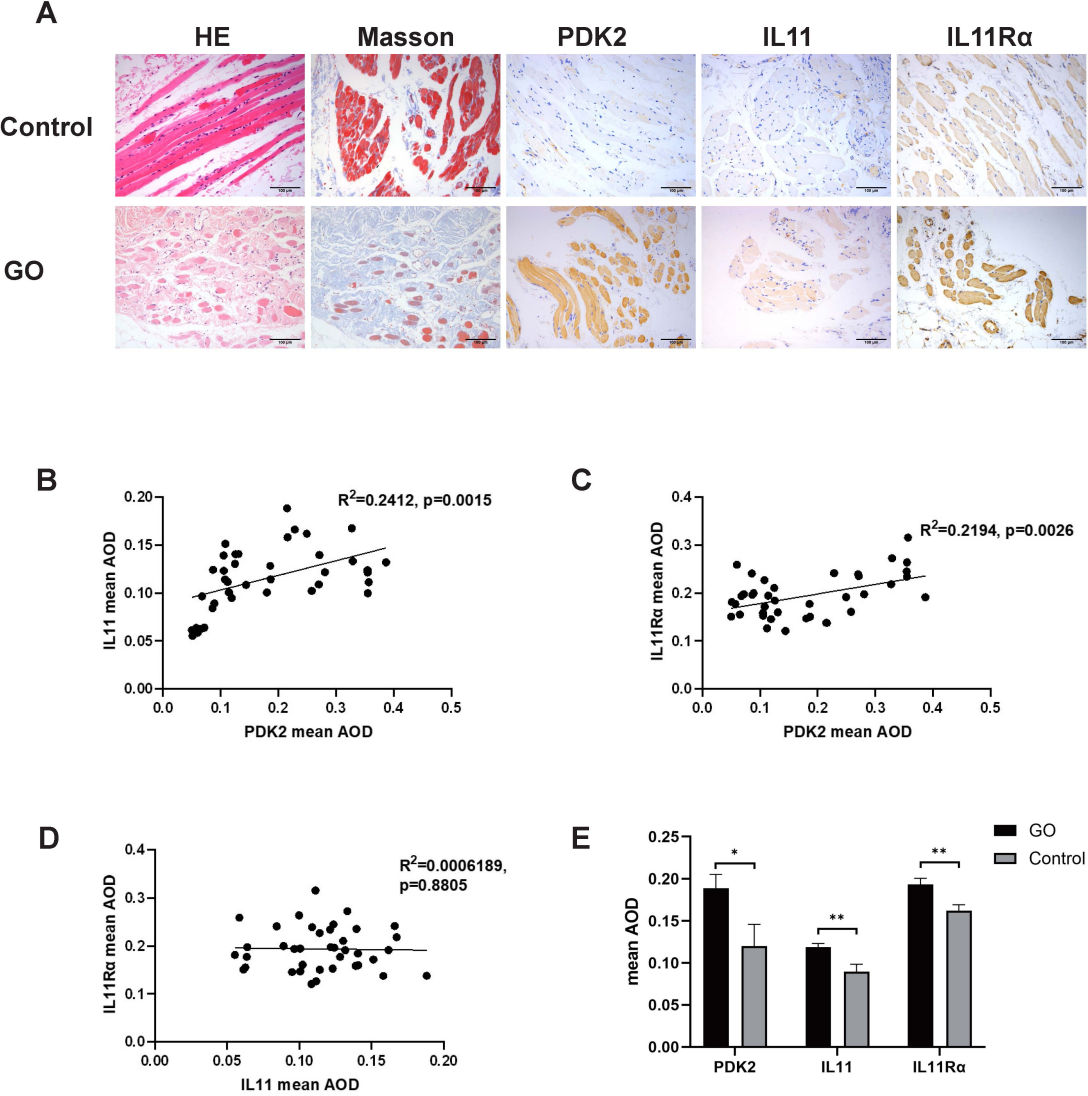
PDK2, IL11, and IL11Rα expression in orbital connective tissues of GO and control patients. **(A)** Representative immunohistochemistry (IHC) staining of collagen (Masson staining, blue), PDK2 (brown), IL11 (brown), and IL11Rα (brown) in the GO group and the control group. Scale bars, 100 μm. **(B)** Average optical density (AOD) was the ratio of integral optical density to area acquired with ImageJ. Correlation between AOD of PDK2 and IL11 (*p*=0.0015). **(C)** Correlation between AOD of PDK2 and IL11Rα (*p*=0.0026). **(D)** Correlation between IL11 of PDK2 and IL11Rα (*p*=0.8805). **(E)** Mean AOD of PDK2, IL11, and IL11Rα in the GO group (n=39) and the control group (n=9). HE, hematoxylin and eosin. Spearman’s test was used for the correlation analysis. Welch’s t-test was used to compare the mean AOD between the GO group and the control group. *p<0.05; **p<0.01.

Histological staining images were analyzed to compare collagen (Masson staining, blue), PDK2 (brown), IL11 (brown), and IL11Rα (brown) expression. As shown in **Figure 1A**, PDK2, IL11, and IL11Rα expression was augmented in the GO group. The average optical density (AOD) of PDK2 in GO group varied from 0.050 to 0.387. AOD in the control group varied from 0.091 to 0.290. AOD of IL11 in the GO group ranged from 0.055 to 0.188 while that in the control group ranged from 0.074 to 0.122. As for IL11Rα, AOD was 0.121–0.316 in the GO group and 0.117–0.187 in the control group. Consistent with previous views (31), collagen expression was elevated in GO extraocular muscles (**Figure 1A**), which contributes to the occurrence of GO myopathy. Correlation analysis among PDK2, IL11, and IL11Rα in the GO group indicated a significant positive correlation between PDK2 and IL11, same as PDK2 and IL11Rα (**Figures 1B, C**). However, analysis between IL11 and IL11Rα showed no significant relativity between IL11 and IL11Rα (**Figure 1D**). The expression levels of three markers in the GO group were significantly higher than those in the control group (**Figure 1E**). These results demonstrate that IL11 signaling may contribute to pathogenesis of GO fibrosis through PDK2-related glycolysis-facilitated pathway.

### 3.2 Suppression of glycolysis inhibits fibrosis in GO OFs

**Figure 2:**
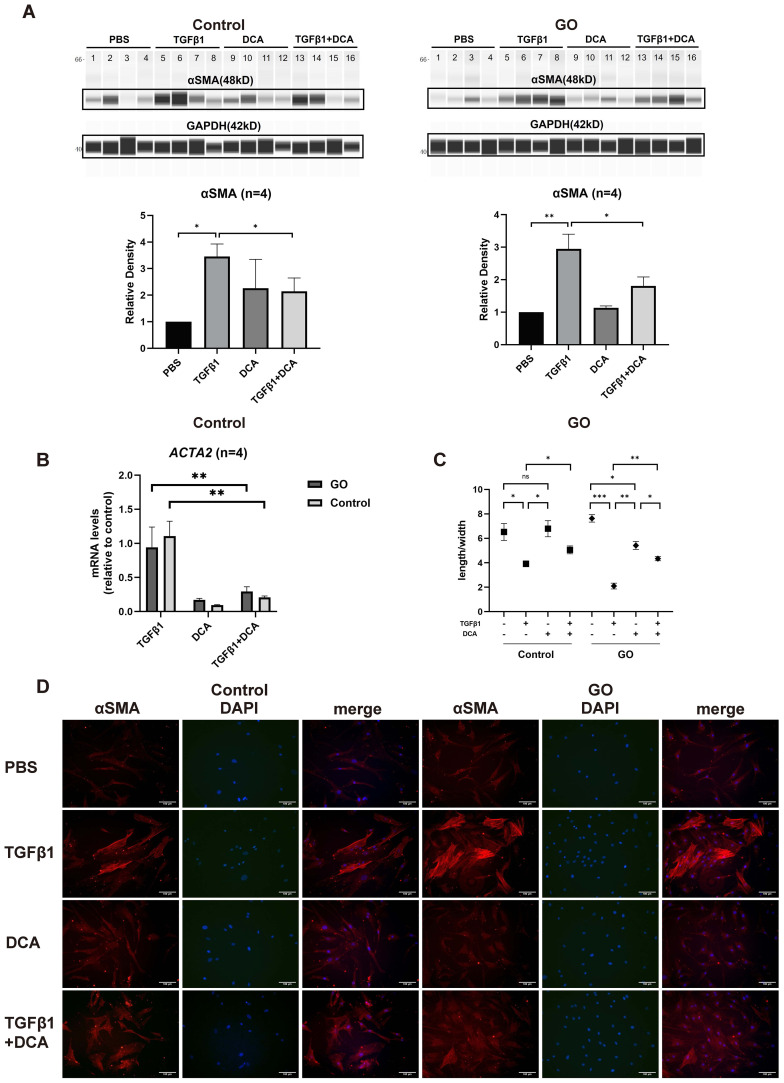
Glycolysis inhibition suppresses fibrosis in GO OFs. **(A)** The representative virtual panels of WES chemiluminescence assay to quantify αSMA and GAPDH after treated with phosphate buffer saline (PBS), TGFβ1 (10ng/ml), DCA (5mM) or TGFβ1 (10ng/ml) + DCA (5mM) for 48 hours in OFs of GO or control groups. Lanes 1,5,9,13 represents one cell strain; lanes 2,6,10,14 represents a second cell strain; lanes 3,7,11,15 represents a third cell strain; lanes 4,8,12,16 represents a forth cell strain. The αSMA protein levels were quantified by the signal intensity in the virtual panels and normalized to GAPDH in each protein sample. The protein levels were shown relative to that of control OFs in the PBS group. **(B)** Comparison of *ACTA2* mRNA levels in the GO and control OFs treated with TGFβ1 (10ng/ml), DCA (5mM) or TGFβ1 (10ng/ml) + DCA (5mM) for 24 hours. The mRNA levels were normalized to the data of control OFs treated with TGFβ1. **(C)** Changes in length to width ratio of OFs. **(D)** The typical images of cytoplasmic αSMA (red) and DAPI (blue) immunofluorescence staining under different treatment. Scale bars, 100μm. (10000 cells per well in 24-well plate) *P<0.05; **P<0.01; ***P<0.001; *n* indicates the number of cell strains in each experimental group. Paired t test was applied to compare the data.

TGFβ1 is the most critical fibrotic cytokine in GO. We stimulated OFs with human recombinant TGFβ1, and αSMA expression was elevated in both GO and control groups (**Figure 2A**), consistent with our pervious study (9, 10). In the following experiments, DCA was adapted as a glycolysis inhibitor. After incubation with TGFβ1 (10 mg/ml) ± DCA (5 mM) for 48 h, αSMA (*ACTA2*) expression was measured. The results demonstrated a significantly decreased αSMA expression when DCA was added at the protein level and mRNA level in both GO groups and control groups compared with OFs stimulated with TGFβ1 alone (**Figures 2B**). When OFs differentiate into myofibroblasts, their length-to-width ratio decreases. Immunofluorescence staining and cell morphological change also showed the same result (**Figures 2C, D**). These results suggest that suppression of glycolysis can inhibit TGFβ1-induced fibrosis of OFs.

### 3.3 PDK2-enhanced glycolysis promotes fibrosis in GO OFs

**Figure 3:**
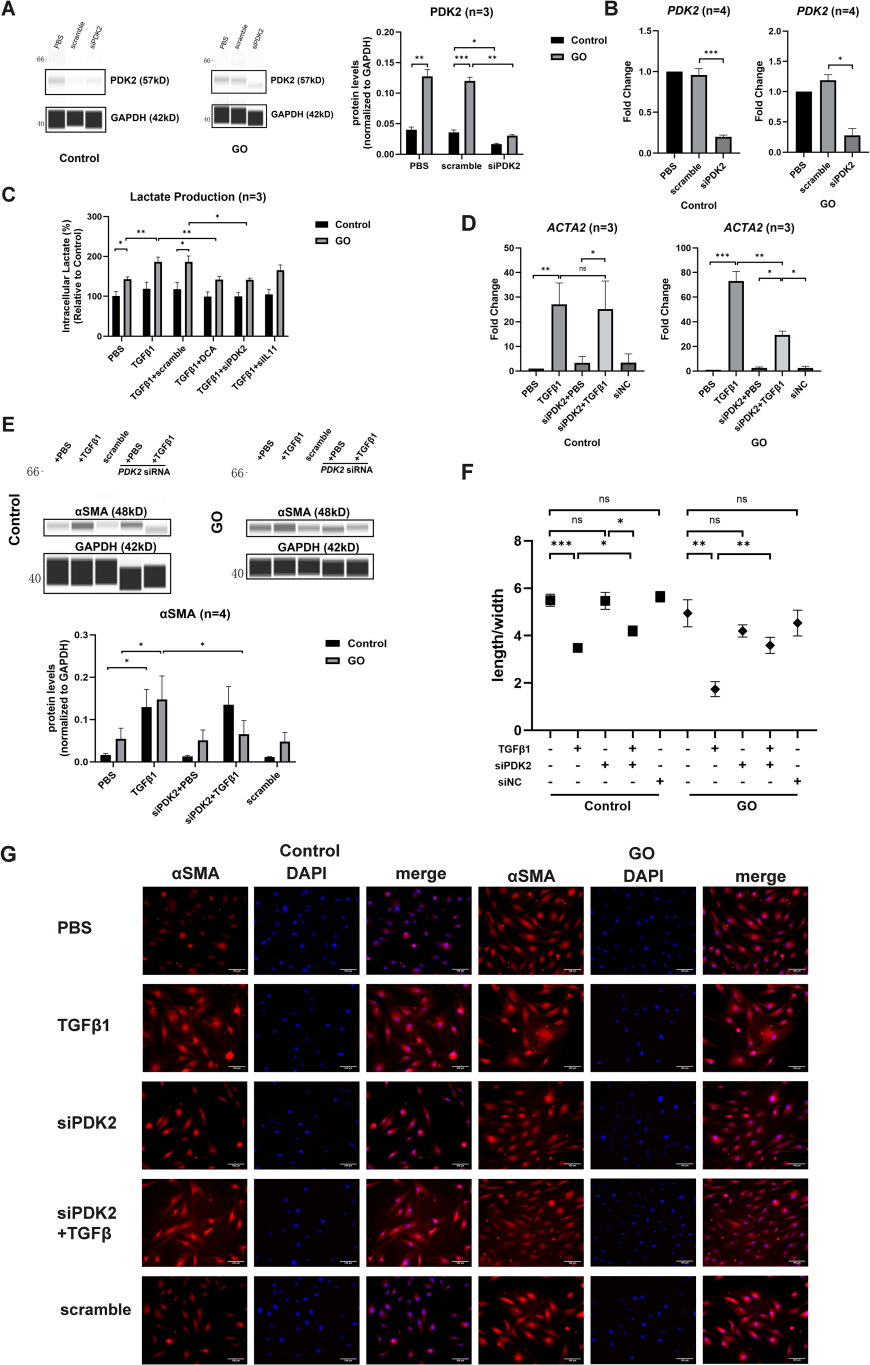
*PDK2* knockdown inhibits glycolysis and fibrosis in the GO OFs. **(A)** Verification of *PDK2* knockdown by WES chemiluminescence assay in the GO and control OFs. The *PDK2* protein levels were quantified by the signal intensity in the virtual panels and normalized to GAPDH in each protein sample. The protein levels were shown relative to that of control OFs in the PBS group. **(B)** Verification of *PDK2* knockdown by quantitative PCR in the GO and control OFs. The *PDK2* mRNA levels were quantified in the PBS, scramble and siPDK2 (*PDK2* siRNA) groups and normalized to the data of control OFs in the PBS group. **(C)** The effects of different treatment on intracellular lactate production in the GO and control OFs. The lactate concentration were expressed as percentage to the data of control OFs in the PBS group. Approximately 2 × 10^6^ cells in each groups were harvested for lactate measurement. The OFs were transfected with *PDK2* or *IL11* siRNA for 12 hours. PBS, TGFβ1 (10ng/ml), or TGFβ1 (10ng/ml) + DCA (5mM) were given after liquid change and acted for 12 hours. **(D)** *ACTA2* mRNA levels after different treatments were tested with quantitative PCR in the GO and control groups. **(E)** The representative virtual panels of WES chemiluminescence assay to quantify αSMA and GAPDH after treated with TGFβ1 (10ng/ml), siPDK2, TGFβ1 (10ng/ml) + siPDK2. **(F)** Changes in length to width ratio of OFs. **(G)** Immunofluorescence of αSMA (red) and DAPI (blue) of OFs. Scale bars, 100μm. *P<0.05; **P<0.01; ***P<0.001; *n* indicates the number of cell strains in each experimental group. Paired t test was applied to compare the data.

The effects of PDK2 on glycolysis were explored in our previous study (9). Quantitative PCR and WES assay confirmed that 12-h transfection and translation with *PDK2* siRNA significantly suppressed *PDK2* transcription in both GO and control OFs (**Figures 3A, B**). Knockdown of *PDK2* or *IL11* or addition of DCA resulted in significantly decreased lactate production comparing with incubation with TGFβ1 alone in GO OFs. However, lactate production showed no significant change in control OFs (**Figure 3C**). Then, we compared fibrotic effect under a different treatment. Knockdown of *PDK2* exerted inhibitive effects on the profibrotic effects of TGFβ1 in GO OFs at both mRNA and protein levels (**Figures 3D, E**). Immunofluorescence and length-to-width ratio revealed decreased αSMA expression and inhibited cell morphological change (**Figures 3F, G**). Collectively, the above results confirmed that *PDK2*-enhanced glycolysis can promote fibrosis in GO OFs.

### 3.4 Enhanced glycolysis facilitates IL11 expression in GO OFs

**Figure 4:**
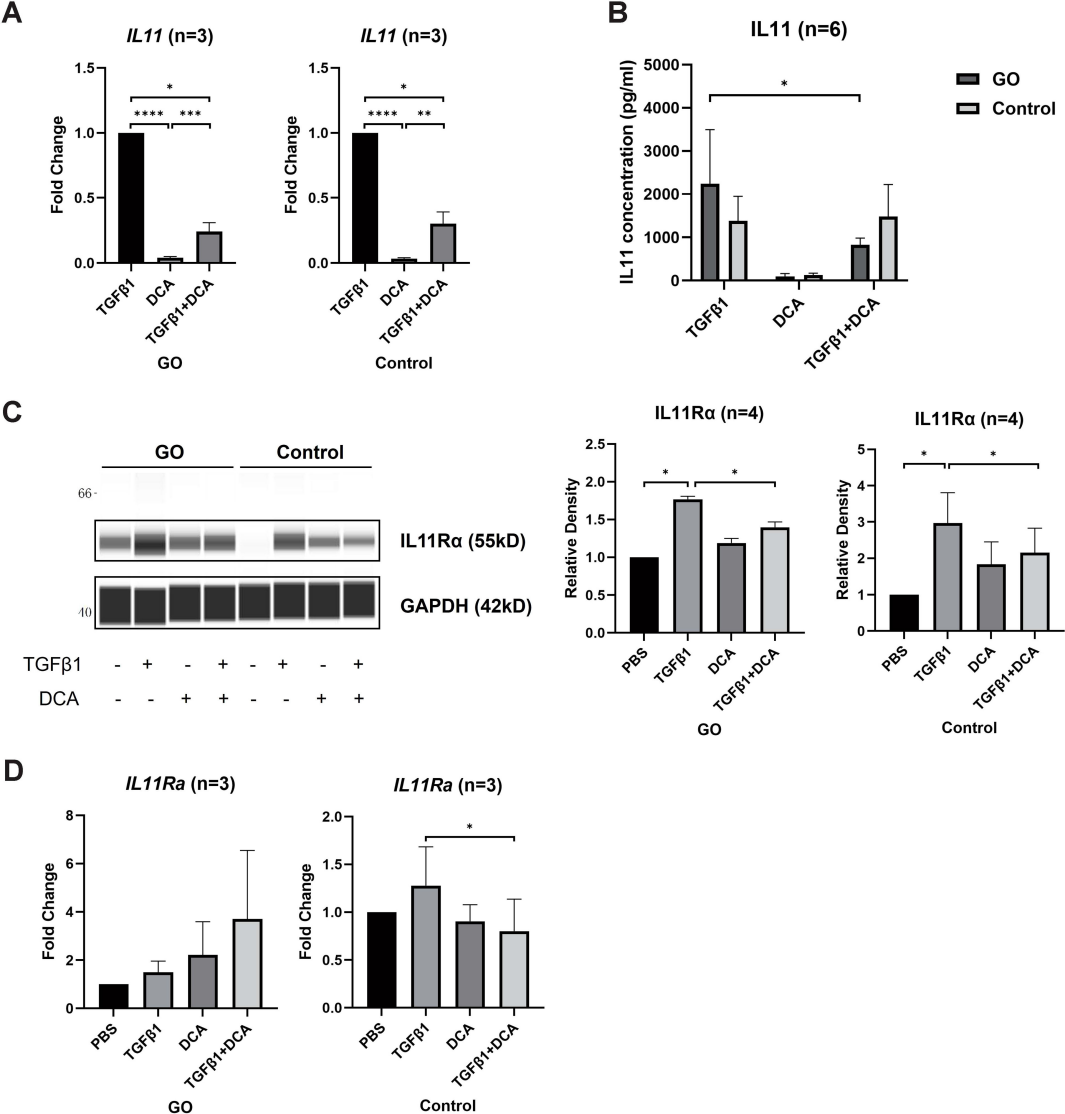
DCA decreases IL11 expression. **(A)** Comparison of *IL11* mRNA levels with 24-h treatment of TGFβ1 (10 ng/ml), DCA (5 mM), or TGFβ1 (10 ng/ml) + DCA (5 mM) detected by quantitative PCR. **(B)** Supernatant IL11 concentration tested by ELISA in different treatment groups. **(C, D)** IL11Rα expression at mRNA level and protein level detected by WES and quantitative PCR. The signal intensities were quantified in virtual panels generated by WES assay and normalized to GAPDH. Lanes 1, 5, 9, and 13 represent one cell strain; lanes 2, 6, 10, and 14 represent a second cell strain; lanes 3, 7, 11, and 15 represent a third cell strain; lanes 4, 8, 12, and 16 represents a fourth cell strain. *p<0.05; **p<0.01; ***p<0.001; *n* indicates the number of cell strains in each experimental group. Paired t-test was applied to compare the data. ****P<0.001.

IL11 has been proven to induce trans-differentiation of OFs to myofibroblasts (29). To explore whether IL11 is related to fibrotic process promoted by glycolysis, we compared the *IL11* and *IL11Rα* mRNA levels of OFs by RT-qPCR between 48-h TGFβ1 (10 ng/ml) ± DCA (5 mM) treatment in the GO and control groups. The *IL11* mRNA level was significantly lower in OFs treated with TGFβ1 + DCA than those treated with TGFβ1 alone (**Figure 4A**) in both groups. As for protein level, we detected the concentration of IL11 in the supernatant with ELISA, and the result corresponded with mRNA level (**Figure 4B**) in GO groups. While in control groups, cell strains presented inconsistency among each other. In addition, DCA decreased IL11Rα levels in both GO and control groups (**Figure 4C**). However, as for mRNA levels, no such tendency appeared (**Figure 4D**). These results suggest that enhanced glycolytic contributes to elevated IL11 expression.

### 3.5 IL11 promotes fibrosis in GO OFs

**Figure 5:**
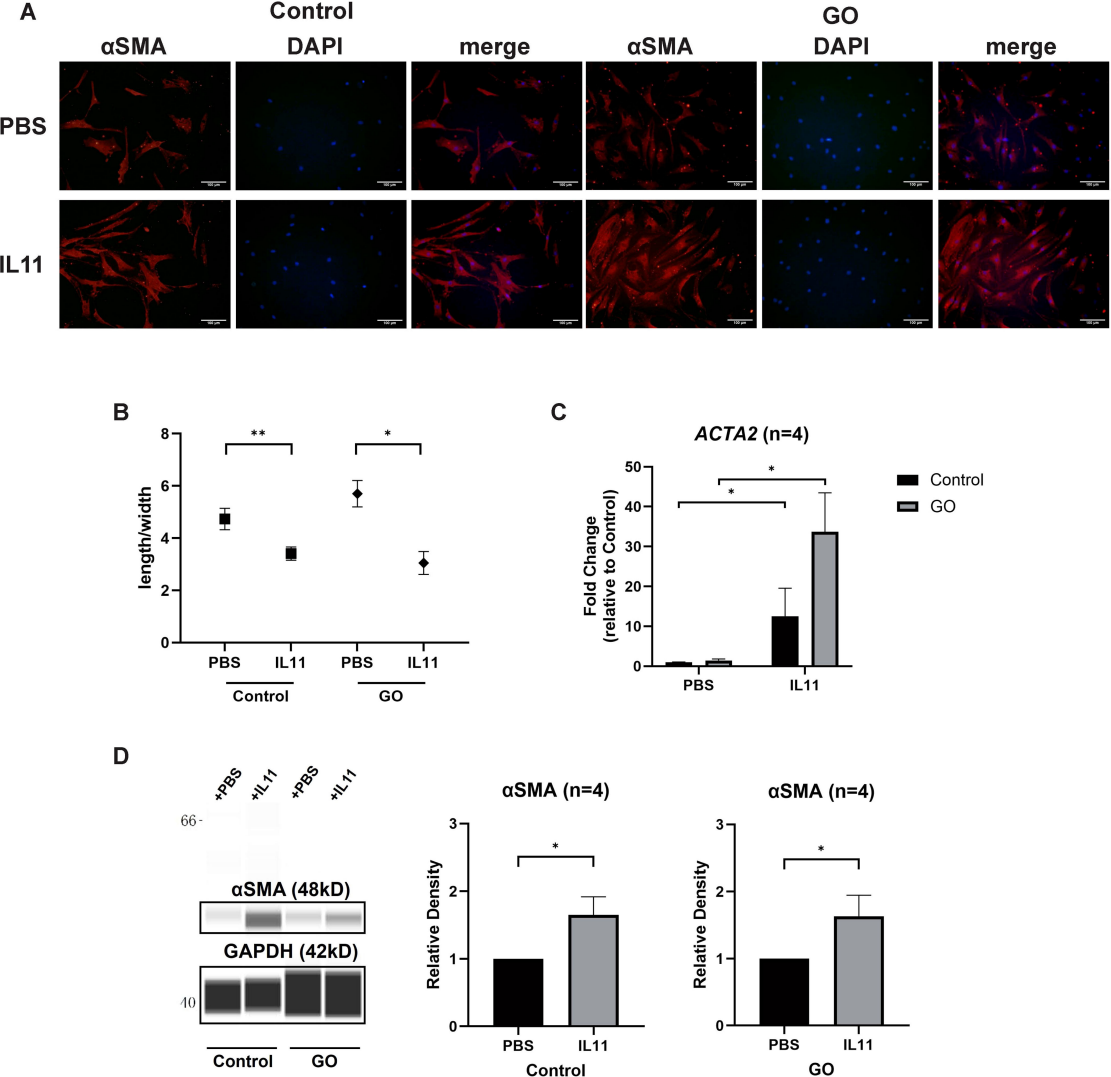
IL11 promotes glycolysis-facilitated fibrosis in GO OFs. **(A)** Immunofluorescence staining of αSMA (red) and DAPI (blue). It shows the effects of IL11 (10 ng/ml) on OFs. Scale bars, 100 μm. **(B)** Changes in length-to-width ratio of OFs. **(C, D)** Virtual panels of WES chemiluminescence assay and quantitative PCR of αSMA (*ACTA2*). The protein levels were quantified by the signal intensity in the virtual panels and normalized to GAPDH. Lanes 1 and 5 represent one cell strain; lanes 2 and 6 represent a second cell strain; lanes 3 and 7 represent a third cell strain; lanes 4 and 8 represent a fourth cell strain. *p<0.05; **p<0.01; *n* indicates the number of cell strains in each experimental group. Paired t-test was applied to compare the data.

To decipher the effect of IL11 on glycolysis-facilitated fibrosis, we compared the differentiation-promoting effect of IL11 to OFs. Immunofluorescence staining and length-to-width ratio revealed that the formation of SMA-positive fibers were increased and OFs’ differentiation with addition of IL11 in both GO and control groups (**Figures 5A, B**). Western blot and RT-qPCR analysis also confirmed that IL11 presented profibrotic effect, which was stronger under conditions of strong glycolysis (**Figures 5C, D**). These results indicate that the IL11 signaling elicits a fibrotic response in OFs and is more pronounced in GO.

### 3.6 IL11 increases sensitivity of GO OFs to TGFβ1

**Figure 6:**
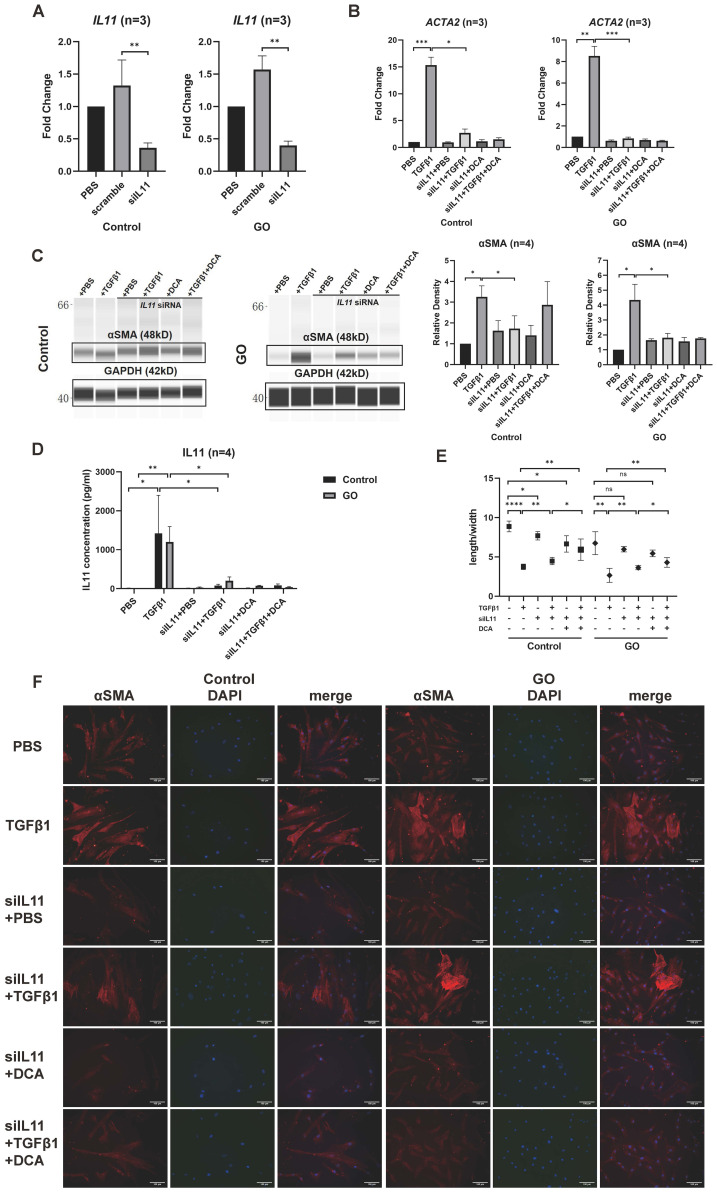
*IL11* knockdown inhibits profibrotic procession in GO OFs. **(A)** Verification of *IL11* knockdown by quantitative PCR in the GO and control OFs. The *IL11* mRNA levels were quantified in the no template control (NTC), negative control (NC), and siIL11 (*IL11* siRNA) groups and normalized to the data of control OFs in the NTC group. **(B)** Quantitative PCR of *ACTA2* mRNA levels in GO and control groups. The data were normalized to the control OFs of PBS group. **(C)** Representative virtual panels of WES chemiluminescence assay to quantify αSMA in GO and control groups. The relative amount of αSMA are quantified by the signal intensity and normalized to GAPDH. **(D)** Supernatant IL11 concentration tested by ELISA in different treatment groups. **(E)** Changes in length to width ratio of OFs. Scale bars, 100 μm. **(F)** Immunofluorescence of αSMA (red) and DAPI (blue) of OFs. *p<0.05; **p<0.01; ***p<0.001; ****p<0.000; *n* indicates the number of cell strains in each experimental group. Paired t-test was applied to compare the data.

The effects of IL11 on TGFβ1 induced myofibroblast differentiation were explored by siRNA transfection. Quantitative PCR of *IL11* siRNA group verified a successful transduction (**Figure 6A**). After knockdown of *IL11*, we stimulated the OFs with TGFβ1 (10 ng/ml), DCA (5 mM), and TGFβ1 (10 ng/ml) + DCA (5mM) for 24 h to test mRNA level and 48 h to test protein level. Although αSMA (*ACTA2*) expression was still elevated, it was significantly reduced compared with that before knockdown in GO OFs (**Figures 6B, C**). The IL11 concentration in supernatant tested by ELISA showed a significant decrease when *IL11* was knocked down (**Figure 6D**). The fluorescence immunostaining and cellular length-to-width ratio also exhibited similar results (**Figures 6E, F**). Interestingly, in several of the control groups, the TGFβ1-induced expression of αSMA was not significantly inhibited. This may be due to the weak glycolytic level in the control group, resulting to a less strong inhibition effect than in the GO group and indicating that IL11 signaling an important component of glycolytic-facilitated fibrosis. Taken together, IL11 increases sensitivity of GO OFs to TGFβ1, which may be a component therapeutic target for GO treatment.

### 3.7 DCA inhibits fibrosis in orthotopic xenografts of the GO orbital tissue

**Figure 7:**
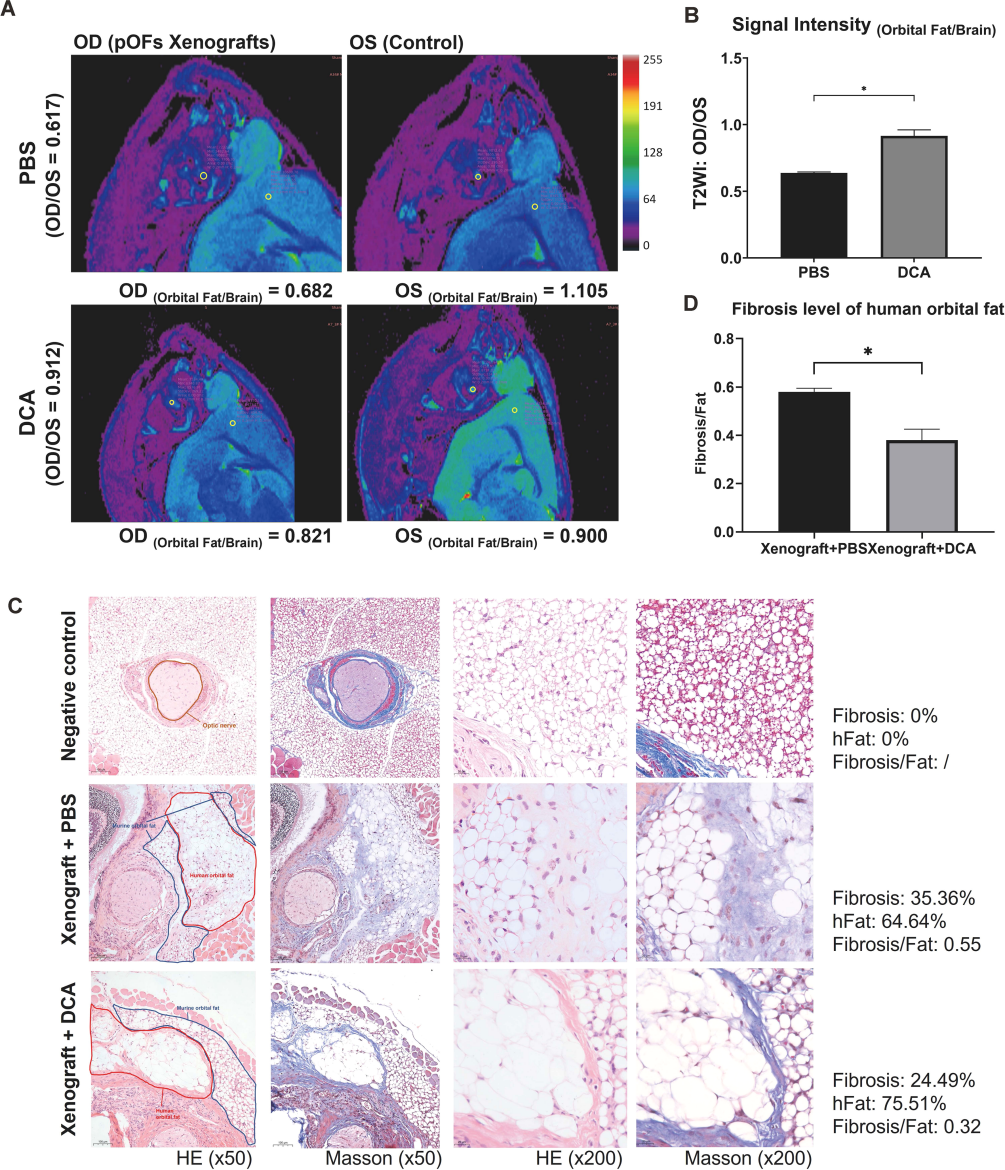
DCA decreases orbital tissue fibrosis *in vivo*. **(A)** Representative pseudo-color images of both orbits of mice. The ROI circles of orbital fat and ipsilateral brain are presented with their signal intensity. OD indicates the right orbit, and OS indicates the left orbit. **(B)** Signal Intensity (Orbital Fat/Brain) of control and DCA groups were statistically compared. *p<0.05; n=5. **(C)** Typical HE and Masson staining images in Matrigel group (control), fibrosis group (xenograft + PBS), and anti-glycolysis group (xenograft + DCA). The human orbital fat (hFat) is circled in red, and murine orbital fat is circled in blue in the first column of images. Scale bars, 100 μm for the first two columns, and 20 μm for the last two columns. **(D)** Fibrosis level of hFat in the fibrosis group (xenograft + PBS) and anti-glycolysis group (xenograft + DCA). *p<0.05; n=3. Paired t-test was applied to compare the data.

We xenografted the GO orbital tissue into orbit of mice to build *in vivo* models. The fibrotic tissues exhibited lower signal intensity compared to control ones (**Figure 7A**), which was consistent with previous studies (32). The results demonstrated that relative signal intensity between the xenografted eye and control eye significantly decreased in the anti-glycolysis group (**Figures 7A, B**). HE staining confirmed the fibrotic effect of the GO orbital tissue orthotopic xenograft into the murine orbit (**Figure 7C**). We compared the ratio of fibrosis area and human orbital fat area in each group, and the result confirmed that inhibition of glycolysis by DCA could restrain orbital connective fibrosis *in vivo* (**Figures 7C, D**).

## 4 Discussion

The metabolic shift in cancer cells towards aerobic glycolysis even in the presence of sufficient oxygen was discovered and named after Dr. Warburg almost a century ago (33). However, the role of metabolic alterations in fibrosis has historically been underappreciated. It was in recent years that aerobic glycolysis had been found existing in several fibrotic diseases (34, 35). Being analogous to cancer, there is a hypothesis speculating the growing requirement for biosynthetic intermediates to support protein synthesis and proliferation being the reason of pathologically enhanced glycolysis in fibrosis (36). TGFβ1 is highly associated with tissue fibrosis. Studies have shown that TGFβ1-induced fibrosis is accompanied by a significant increase in cellular glycolysis levels and reprogramming of glucose metabolism (37, 38). Studies conducted on the inhibition of glycolysis to treat fibrosis has been arising recently (39–41). Increased lactic acid levels and expression of lactate dehydrogenase (LDH) was discovered in lung tissues of IPF patients, suggesting that pyruvate’s entrance into glycolytic pathway can promote fibrosis (42).

With regard to GO, we previously demonstrated that *PDK2* overexpression contributed to the enhanced glycolysis, and DCA could suppress glycolysis to reduce fibroblast proliferation (9). Our conclusion was reinforced by studies using ^18^F-FDG-PET/MRI examination (43) and microarray analysis in GO (44) and proteomic analysis in GD (45). *In vitro* experiments have verified inhibition of aerobic glycolysis suppressing fibroblast activation in the renal interstitium (46). Consistent with past studies, our study demonstrated that both glycolysis inhibitor DCA and knockdown of *PDK2* could suppress TGFβ1-induced OF phenotype switching in both GO patients and healthy controls. In a previous study, serum IL11 levels and IL11 expression in the local orbital connective tissues had been found to correlate with CAS score in GO patients (29). *In vivo* experiments further confirmed this conclusion. Moreover, we showed that IL11 secretion downstream of TGFβ1 was also decreased when glycolysis was blocked in GO OFs. Our immunofluorescence results showed no such significant change in cell morphology when stimulated with IL11 as stimulated by TGFβ1. We, therefore, assumed that IL11 is one of the critical downstream intersections between fibrotic process and glycolysis, which could be a potential therapeutic target for GO.

Our data demonstrated a decreased tendency of lactate production when *IL11* was knocked down with *IL11* siRNA in GO OFs, although the results did not show a statistical difference. We further confirmed that knockdown of *IL11* significantly block OF phenotype switching in both GO and healthy subjects from both mRNA and protein levels. This anti-fibrotic effect was stronger in GO than in control OFs. Such difference was parallel to the previous finding that glycolysis was stronger in GO OFs. IHC analysis also demonstrated a significant positive correlation between the expression of PDK2 and that of IL11. These results provided evidence for our assumption. These findings suggest that targeting aerobic glycolysis could be a viable therapeutic approach for GO. While numerous studies have highlighted the efficacy of DCA in inhibiting aerobic glycolysis and targeting tumor metabolism in both *in vitro* and *in vivo* models (47), its clinical application has been limited by concerns regarding peripheral neurotoxicity and potential carcinogenicity (48). In contrast, IL11, as a downstream effector of aerobic glycolysis, offers greater specificity and a potentially superior safety profile, positioning it as a promising candidate for targeted therapy in GO.

Gp130 is a receptor of significant interest due to its role in mediating multiple signaling pathways. IL11 activates downstream pathways by binding to IL11Rα and forming a homodimer with gp130. Interestingly, gp130 also serves as a receptor for IL6 (49, 50). Numerous studies have highlighted the critical role of IL6 signaling in the pathogenesis of autoimmune diseases, including Graves’ orbitopathy (GO) (51, 52). For instance, the IL6-gp130 signaling axis drives T-cell activation, effector cell differentiation, and TH17 cell expansion (53). Targeting gp130, or promoting its degradation, has been shown to inhibit fibrosis in both *in vitro* and *in vivo* experiments (54, 55). However, gp130 is integral to many physiological processes, including neuronal activity, cardiac protection, immune responses, and wound healing, and it serves as a receptor for multiple cytokines. This broad involvement raises concerns about the specificity and safety of targeting gp130 for therapeutic purposes (56, 57). As such, identifying alternative, more specific targets for antifibrotic therapies remains imperative.

IL11Rα, the ligand of IL11, was found to be existing on the fibroblast’s cell membrane. Fibroblast-to-myofibroblast transition was thought to be dependent on an autocrine IL11 signaling loop in the heart (26), liver (27), kidney (58), and lung (28). *In vivo* experiments on liver fibrosis have confirmed that blocking IL11Rα with a specific antibody effectively inhibits fibrosis (27). There is no doubt that TGFβ1 increases secretion of IL11. However, the effect of TGFβ1 on IL11Rα is not fully explored. According to IHC results, we found that IL11Rα was lesser in tissues of control than GO patients. We also confirmed TGFβ1 inducing IL11Rα synthesis with *in vitro* experiment. Thus, a statistical correlation between IL11 and IL11Rα is expected to be existing. However, correlation analysis showed no correlation between them. *In vitro* experiments showed a rather interesting result. Further observing the data, we could find that IL11Rα in control OFs tended to increase by a higher multiple induced by TGFβ1. This discrepancy suggests that IL11Rα may operate through mechanisms partially independent of IL11, with distinct regulatory pathways being activated by TGFβ1.

Our data also suggest that control OFs, which exhibit lower baseline glycolysis and IL11Rα levels, undergo more significant changes in response to TGFβ1 stimulation compared to GO OFs. This observation aligns with the hypothesis that TGFβ1-induced expression of IL11 and IL11Rα occurs through relatively independent mechanisms. Moreover, while IL11 signaling relies on specific binding to IL11Rα, excessive IL11 production may lead to saturation of IL11Rα, potentially introducing a feedback mechanism that modulates the signaling intensity. The potential positive association between IL11Rα expression and both fibrosis and glycolysis underscores its relevance as a therapeutic target. However, the apparent independence of IL11 and IL11Rα pathways suggests unexplored complexity in their regulation. To elucidate the interplay between glycolysis and fibrosis, comprehensive *in vivo* studies and integrated metabolomic and transcriptomic analyses are essential. These approaches could provide deeper insights into the regulatory mechanisms underpinning TGFβ1-induced changes in IL11 and IL11Rα expression and their implications for fibrosis and glycolysis in GO.

## 5 Conclusion

In conclusion, this study demonstrates that PKD2-enhanced glycolysis is partly responsible for phenotype switching induced by TGFβ1, and IL11 signaling may mediate this process. These findings suggest that the block of IL11 signaling may inhibit fibrosis through downregulating glycolysis, providing novel therapeutic target for GO.

## Data Availability

The raw data supporting the conclusions of this article will be made available by the authors, without undue reservation.
